# Application of the Lomb-Scargle Periodogram to InvestigateHeart Rate Variability during Haemodialysis

**DOI:** 10.1155/2020/8862074

**Published:** 2020-12-08

**Authors:** Jill Stewart, Paul Stewart, Tom Walker, Latha Gullapudi, Mohamed T. Eldehni, Nicholas M. Selby, Maarten W. Taal

**Affiliations:** ^1^School of Health and Social Care, University of Derby, Derby, UK; ^2^Centre for Kidney Research and Innovation, University of Nottingham, Derby, UK; ^3^Renal Unit, Royal Derby Hospital, Derby, UK

## Abstract

Short-term cardiovascular compensatory responses to perturbations in the circulatory system caused by haemodialysis can be investigated by the spectral analysis of heart rate variability, thus providing an important variable for categorising individual patients' response, leading to a more personalised treatment. This is typically accomplished by resampling the irregular heart rate to generate an equidistant time series prior to spectral analysis, but resampling can further distort the data series whose interpretation can already be compromised by the presence of artefacts. The Lomb–Scargle periodogram provides a more direct method of spectral analysis as this method is specifically designed for large, irregularly sampled, and noisy datasets such as those obtained in clinical settings. However, guidelines for preprocessing patient data have been established in combination with equidistant time-series methods and their validity when used in combination with the Lomb–Scargle approach is missing from literature. This paper examines the effect of common preprocessing methods on the Lomb–Scargle power spectral density estimate using both real and synthetic heart rate data and will show that many common techniques for identifying and editing suspect data points, particularly interpolation and replacement, will distort the resulting power spectrum potentially misleading clinical interpretations of the results. Other methods are proposed and evaluated for use with the Lomb–Scargle approach leading to the main finding that suspicious data points should be excluded rather than edited, and where required, denoising of the heart rate signal can be reliably accomplished by empirical mode decomposition. Some additional methods were found to be particularly helpful when used in conjunction with the Lomb–Scargle periodogram, such as the use of a false alarm probability metric to establish whether spectral estimates are valid and help automate the assessment of valid heart rate records, potentially leading to greater use of this powerful technique in a clinical setting.

## 1. Introduction

Patients receiving chronic haemodialysis (HD) as a result of end-stage kidney disease (ESKD) are at a much higher risk of morbidity and mortality [1]. The prevalence of cardiac complications in this population is (in part) because HD causes circulatory stress leading to abnormal haemodynamic and cardiovascular function [2]. While not fully explored, it appears that cardiovascular regulatory mechanisms are unable to adequately compensate for fluid removal from the vascular compartment during HD [2]. The strong, bidirectional, and complex relationships between the kidney and heart can be investigated via the analysis of heart rate variability (HRV) in order to provide valuable insight into physiological and pathological conditions and to enhance risk stratification [3, 4].

Cardiac activity is controlled by the sympathetic (accelerating) and parasympathetic (decelerating) arms of the autonomic nervous system (ANS) which induce oscillations between successive sinus beats at different rhythms. These can be quantified on the electrocardiograph (ECG) as the interval between the peak of one “QRS” complex to the peak of the next, referred to as the “RR” interval. Analysis of HRV rests upon different mathematical (time-domain) and spectral (frequency-domain) measures that have identified significant physiological rhythms hidden in RR interval fluctuations, oscillating at specific frequencies [3]. These rhythms can be characterised by the signal energy (power) found in a low frequency (LF) band (0.04 < LF < 0.15 Hz) and a high frequency (HF) band (0.15 < HF < 0.4 Hz). The power component in the HF band is correlated with parasympathetic activity [5] and corresponds to the HR variations related to the respiratory cycle. Power in the LF band involves contributions from both sympathetic and parasympathetic activity, and it has been suggested that a better approach to understanding sympathetic activity relies on analysing the LF/HF ratio [3, 5]. As shown in previous studies [5], the spectral parameters of HRV can describe and categorise patients individual response to HD and could potentially predict morbidities, for example, intradialytic hypotension.

The power content of the LF and HF frequency bands is computed via the power spectral density (PSD) estimate of the RR tachogram, most commonly using a fast Fourier transform (FFT) [4]. While straightforward and rapid, FFT requires artificial interpolation of the time-varying heart rate to satisfy the axiomatic requirement of a time-invariant sampling rate. Resampling, in effect a nonlinear low-pass filter, also makes an implicit assumption about the form of underlying variation in the data series. Autoregressive- (AR-) based periodograms have also been employed as they can use shorter segments of data without losing spectral resolution [6]. However, in addition to requiring an evenly sampled data series, AR techniques are complex to implement and highly dependent on the choice of model or model order [4].

With both methods, it is recommended [3] to visually inspect ECG data and, if necessary, correct it prior to HRV analysis to minimize any interference that may compromise results [7]. This is highly impractical for large datasets obtained in clinical studies

- Which are significant in volume per patient HD treatment, involving multiple recordings of 4 hours in duration
- Which suffer from noise from a variety of sources such as the influence of electromagnetic interference [8] and artefacts due to patient movement
- Which have missing data due to a loss of signal (for example, if a patient became unwell or otherwise took a break from monitoring during treatment)

It is common for HD patients to experience a significant number of ectopic beats (cardiac dysrhythmia) during dialysis [9], which further complicates the analysis.

A lesser known but more potentially more convenient approach to spectral analysis is the Lomb–Scargle (LS) method [10] where time-varying data are weighted on a point-by-point basis, rather than on a per-time basis, thus avoiding the requirement to resample data. This method is equivalent to AR and FFT in the case of equally-spaced observations [6, 11], but the LS periodogram is less likely to introduce spurious frequencies [4, 12, 13] and “jitter” [6] to the power spectrum when noise is added to the signal. For these reasons, the LS periodogram is potentially a more robust technique for use within a clinical setting, such as the present study application.

This study application is the iTrend (Intelligent Technologies for Renal Dialysis) programme, a long-term collaborative project conducted by a multidisciplinary research team from the Universities of Derby and Nottingham and the Royal Derby Hospital Renal Unit in the UK. The primary goal of the programme is to develop supporting technologies to enable personalised treatment in ESKD [14]. Adult participants were recruited from the Renal Unit's prevalent dialysis population and received continuous noninvasive monitoring of heart rate via ECG and haemodynamic parameters using pulse wave analysis (Finapres NOVA) during entire dialysis treatments. The protocol was approved by the West Midlands Research Ethics Committee and participants gave written informed consent.

Before analysis of patient data can be attempted, it is first necessary to understand the effects of data processing. Most guidelines and practice [3] have been developed for equidistant time series data (e.g., FFT), and it is unclear if these approaches are useful with LS which is able to work on the uncorrected and irregular time series of RR intervals. The purpose of this paper is to establish a reliable method of HRV analysis suitable for large datasets such as those obtained in the clinical setting of the iTrend project. A number of techniques commonly used in the preprocessing of RR interval tachograms prior to spectral analysis are evaluated in order to establish which combination of methods is the most reliable and most practical for use with clinical data. This paper will evaluate the effects of RR interval correction on a well-defined synthetic data series and then on sample patient data. This work addresses a need to understand the effects of signal processing on the interpretation of spectral parameters in order to better discriminate between those influenced by the patient state and those generated by the algorithm.

## 2. Materials and Methods

### 2.1. Lomb–Scargle Power Spectral Density Estimation

The Lomb–Scargle approach, originally derived for astrophysics applications, is a well-defined procedure to generate a power spectrum to detect and characterise the periodic components of a signal. Time-varying data are weighted on a point-by-point basis, rather than on a per-time basis, thus avoiding the requirement to resample data. Previous work has shown that LS outperforms FFT and AR methods when noise and ectopic beats are present in the RR tachogram [12], which suggests that LS may be more suitable for use within a clinical setting.

For the time series *x* [*t*_*n*_] which is precentred around the mean, the normalised LS periodogram is defined as

(1) $$\begin{array}{c} P_{xx}\left(f\right)=\frac{1}{2\sigma^{2}}\left(\frac{{\left[\sum_{n=1}^{N}\left(x\left[t_{n}\right]-\bar{x}\right)\cos\left(2\pi f\left[t_{n}-\tau \right]\right)\right]}^{2}}{\sum_{n=1}^{N}\cos^{2}\left(2\pi f\left[t_{n}-\tau \right]\right)}+\frac{{\left[\sum_{n=1}^{N}\left(x\left[t_{n}\right]-\bar{x}\right)\cos\left(2\pi f\left[t_{n}-\tau \right]\right)\right]}^{2}}{\sum_{n=1}^{N}\sin^{2}\left(2\pi f\left[t_{n}-\tau \right]\right)}\right), \end{array}$$

where *x* and *σ*^2^ are the mean and variance of the time series. The sine and cosine coefficients are normalised separately by a frequency-dependent time delay, *τ*, in order to make the transformation insensitive to time shifts in the data.

(2) $$\begin{array}{c} \tau =\tan\left(4\pi f\tau \right)=\frac{\sum_{n=1}^{N}\sin\left(4\pi ft_{n}\right)}{\sum_{n=1}^{N}\cos\left(4\pi ft_{n}\right)}. \end{array}$$

The shortest period over which HRV metrics should be assessed is five minutes [3], so the lowest frequency that can be resolved is 1/300 = 0.003 Hz. Guidelines [3] further specify the upper frequency limit of the HF band is 0.4 Hz. This leads to a constraint that the minimum (*N*) number of points within the five-minute segment is 240.

There are some practical considerations that should be made when analysing unevenly sampled data relating to the choice of frequency limits and the grid spacing. The lower limit is well-defined as the fundamental frequency *f*_0_ of a sine wave of period equal to the whole interval *T* and so it is set by the sampling duration. The highest frequency that can be coded at a given sampling rate, the Nyquist frequency, is defined as *f*_*c*_=1/2Δ*T*. However, VanderPlas [15] shows that the sampling interval for the time-varying LS method, Δ*T*=*T*/*N*, tends to be greater than any limits on the time-invariant case, so it is more appropriate to set a pseudo-Nyquist frequency based on the precision of the time measurements as *f*_*c*_′=1/2*p* where *p* is the largest value such that each spacing Δ*t*_*i*_ is an integer multiple of this factor.

The choice of how finely to sample the frequencies is less obvious for unevenly sampled data and is a balance between too fine a grid with long computation times and too coarse a grid whose spacings are larger than the expected width of the peaks. Data observed through a rectangular window of length *T* will have sinc-shaped peaks of width 1/*T*, so in order to ensure the grid captures each peak, equation (1) should be implemented with some oversampling factors. VanderPlas [15] recommends a grid size of Δ*f*=1/5*T*.

A different type of frequency limit exists where observations consist of short-duration integrations of a continuous signal. Each observation is effectively a convolution of the underlying ECG signal with a rectangular function of *δt*. This leads to a “window” limit of *f*_max_*∞*1/2*δt*, beyond which signals are attenuated to zero [15]. The constant of proportionality depends on the shape of the window describing individual observations, and for the RR tachogram, the windowing function is a series of very narrow spikes. By analogy, this gives a maximum frequency of

(3) $$\begin{array}{c} f_{\max}=\frac{1}{T}\frac{1}{2\delta t}=\frac{f_{s}}{2T}. \end{array}$$

Provided that these conditions are met (the segment must be five minutes in duration, it must contain a minimum of 240 data points, the maximum frequency must lie beyond 0.4 Hz, and the precision of measurements must be at most 0.625 seconds) the spectral analysis can be performed. The mean value of the RR series should be subtracted to avoid the effect of high energy values distorting the frequency of the spectrum prior to performing any power spectral analysis. In a real (as opposed to a synthetic) dataset, some detrending method should also be used to suppress drifts in the underlying ECG signal.

### 2.2. Synthetic Data Series

In order to separate the effect of signal processing techniques from real features of the underlying data, a simple synthetic signal with well-defined properties was generated following the method described by Clifford [12]. An artificial tachogram was generated by mixing two sine waves with frequencies at the centre of the LF and HF bands (*ω*_*l*_ = 0.095 Hz, *ω*_*h*_ = 0.275 Hz) [12]. The LF component was given an amplitude of *A*_*l*_ = 2 bpm, and the HF band is given a larger amplitude *A*_*h*_ = 2.5 bpm (it will be seen later that RR correction can filter the HF components, so it was emphasised in the synthetic data series). The average heart rate was set at HR_0_ = 60 bpm.

(4) $$\begin{array}{c} \text{HR}\left(t\right)={\text{HR}}_{0}+A_{l}\sin\left(\omega_{l}t\right)+A_{h}\sin\left(\omega_{h}t\right), \end{array}$$

(5) $$\begin{array}{c} \text{RR}\left(t\right)=\frac{60}{\text{HR}\left(t\right)}. \end{array}$$

**Figure 1:**
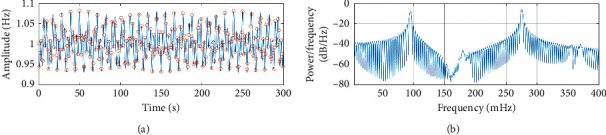
Synthetic data series with mean frequency of 1 Hz and zero phase, realized from the sum of two sinusoids via equation (4) with RR intervals (shown as circles) generated from (5) [12]. The resulting LS periodogram accurately locates peaks at *ω*_*l*_ = 0.095 Hz and *ω*_*h*_ = 0.275 Hz. (a) Synthetic signal. (b) Lomb–Scargle power spectral density estimate.

The first result in the RR tachogram is defined as the first RR interval (RR_1_ at *t*_1_). The next RR interval (RR_2_ at *t*_2_) is defined where the RR value equals the time difference between *t*_2_ and *t*_1_ [12]. This is generalised as (6), and the result is shown in Figure 1.

(6) $$\begin{array}{c} {\text{RR}}_{n}\geq t_{n}-t_{n-1}. \end{array}$$

The synthetic signal was then distorted by adding zero-mean white Gaussian noise to equation (4), which is thought to be representative of the type of noise encountered in real ECG data [16]. We did not model the effects of 50 Hz power interference as this is adequately filtered by both the hardware and then the software processing of the ECG signal [17]. The superimposed noise signal was scaled to match the standard deviation of the real biological signal (0.228) which is described later in this paper. As the LS periodogram is weighted on a per point basis, it has the unique property of being able to provide a spectral estimate where a data point is missing, and so a number of data points were discarded at random from the RR tachogram (6). This synthetic data series was used to explore the limits of the LS periodogram and to understand the effects of signal preprocessing on the PSD estimate.

**Figure 2:**
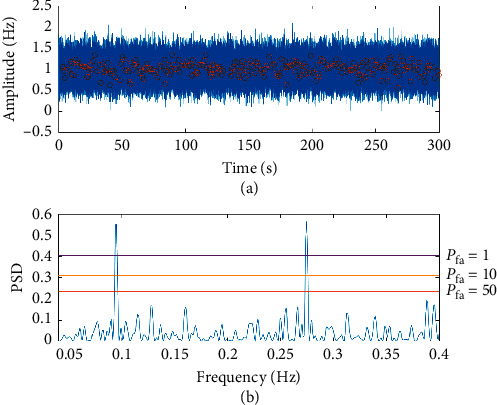
Signal masked by noise (solid line in upper graph) with 20% of RR intervals discarded at random (the remaining RR intervals appear as circles in upper graph). The frequencies of the original sinusoid are both accurately located at *ω*_*l*_ = 0.095 Hz and *ω*_*h*_ = 0.275 Hz and are the only peaks to exceed a FAP of 50% in the resulting periodogram. (a) Synthetic signal. (b) Lomb–Scargle spectrum.

The distorted signal and its power spectrum are shown in Figure 2. The magnitude of each peak depends on the number of observations and the signal-to-noise (SNR) ratio. In the case of few observations and high levels of noise, the spurious background peaks can become comparable in magnitude to the true peaks. This effect can be quantified by the calculation of false alarm probability (FAP) which is the probability that a dataset with no signal would, due to random error, lead to peaks of a similar size. If the expected peak width is *δf*=1/*T*, then the number of independent frequencies (peaks) in a range 0 ≤ *f* ≤ *f*_max_ is assumed to be *N*_eff_ = *f*_max_*T* [15]. FAP is then estimated as

(7) $$\begin{array}{c} \text{FAP}\left(z\right)\approx 1-{\left[P_{\text{single}}\left(z\right)\right]}^{N_{\text{eff}}}. \end{array}$$

This important but neglected evaluation step provides the second opportunity to decide whether to include or exclude PSD estimates from further analysis. Any PSD estimate lacking at least one peak above a FAP of 50% can be rejected on the basis that it cannot discriminate signal from noise.

### 2.3. The Effects of RR Editing on HRV Analysis

Other than noise, artefacts in real data would include abnormal heart beats with unusual timing. For example, unusually short RR intervals (ectopic beats) will introduce higher than normal frequency components, causing an overestimation of HF power. Missing data would emphasise longer RR intervals and cause a bias towards LF power. Any clinical signal could suffer from these effects, so the implication is that the signal would require some form of preprocessing to identify and correct doubtful points [3].

A great deal of methodological diversity is seen in the preprocessing of data prior to analysis. Aberrant RR intervals are most commonly identified in comparison to a range of expected values based on previous RR intervals [18–21] or by comparison with a statistical measure of the whole RR tachogram [7, 22]. In this work, false beats are initially detected using the approach that seems most commonly used where a high (+32.5%) or low (−24.5%) threshold for the relative variation in successive RR intervals is exceeded [18, 19]. It will be shown that this approach biases the error selection and distorts the PSD, and so a symmetric criterion of ±10% will be proposed and tested.

The guidance regarding which techniques are most suitable for correcting aberrant RR intervals is also diverse. Methods that exclude outlier values can lead to a systemic loss of information in time-invariant data [23]. Methods to replace outlier values with average values [24, 25] or interpolation [26] can change the power of the frequency components in spectral analysis by introducing false shapes [27]. The effect of any of these approaches when used in conjunction with time-varying PSD estimation is missing from literature. To understand the role of RR editing with the LS periodogram more fully, comparisons were made between five different correction methods. Three of these methods are commonly used with equidistant time-series HRV data (methods 1, 2, and 3), one method has been specifically proposed for use with the LS approach (method 4), and a final method is used with the LS periodogram here for the first time (method 5).

#### 2.3.1. Method 1: Exclusion of Suspect Data Points (LS Baseline)

Rather than discarding data at random, only those intervals flagged as in error [19] (on average 15% of the RR intervals over 100 runs) are excluded from the LS estimation. This method will provide a baseline against which other methods are compared and would be the preferred method as no further preprocessing of the signal is required, thus limiting the potential for spectral analysis to be distorted. This method of exclusion of suspect data points from spectral analysis is no longer recommended for use with equidistant time-series methods [13].

#### 2.3.2. Method 2: Rules-Based Editing (Rules)

An interval that is identified as incorrect will be further analysed in combination with its neighbours and retained if it can be identified as forming part of a physiologically plausible pattern [28]. Otherwise, the aberrant RR interval can be corrected by

- Summing with one or more neighbouring intervals, which would apply in the case of a false trigger occurring between normal beats.
- Dividing one large interval into two or more intervals of acceptable size, which would apply (for example) in the case of missed heart beats.
- Adding two or more intervals and dividing the sum into two or more acceptable values. This would occur (or example) when an ectopic beat occurs in the place of a normal (sinus) beat.

The selected correction would be the one that brings the new RR interval closest to the mean average of RR intervals as calculated from the previous minute of data [28].

#### 2.3.3. Method 3: Replacement

All intervals identified as being in error were removed from the HRV tachogram and replaced. The replacement was either by an interpolated value (linear or cubic spline) using 3 previous and 1 following neighbour [8] or else by the mean average of the previous 60 seconds of RR intervals [28].

#### 2.3.4. Method 4: Ornstein–Uhlenbeck Third-Order Gaussian Process Filtering (OUGP)

The OUGP filter is a reduced form of a Wiener filter, where a one-dimensional series of measurements as a function of time can be solved more efficiently as its inverse matrix via a tridiagonal system of equations. Full details and derivation can be found in [29] but are summarised for a series of measurements *x*_*j*_ and time *t*_*j*_, *j* = 1,…, *n*, as

(8) $$\begin{array}{c} \underset{j}{\sum }T_{ij}u_{j}=\left\{\begin{array}{cc} \frac{\left(x_{1}-x_{2}\right)}{2w_{1}}, & i=1,\\ \frac{\left(x_{i}-x_{i+1}\right)}{2w_{i}}+\frac{\left(x_{i}-x_{i-1}\right)}{2w_{i}}, & i=2,\ldots ,n-1,\\ \frac{\left(x_{n}-x_{n-1}\right)}{2w_{n-1}}, & i=n, \end{array}\right. \end{array}$$

where ∑_*j*_*T*_*ij*_*u*_*j*_ is the sparse tridiagonal system convolving the matrix *T* and the output of a filtered sequence *u*. The input *w* is complex, but the filter is taken to be the real part of the result as

(9) $$\begin{array}{c} Hy=ℜ\left(u\right),\\ Ly=y-ℜ\left(u\right). \end{array}$$

The OUGP method is implemented here as a filter passing frequencies in the band 0.003–0.4 Hz. When applied to an unevenly sampled RR tachogram, the OUGP filter exhibits a stable third-order zero-phase frequency response with explicit −3 dB points [30], leading to a recommendation that that it would be suitable for implementation in conjunction with LS periodogram (which motivated its inclusion here).

#### 2.3.5. Method 5: Denoising by Empirical Mode Decomposition (EMD)

It is a data-driven method to denoise nonlinear and nonstationary multicomponent time series, *x* (*t*), by decomposing it into a finite number of signal-dependent semiorthogonal zero-mean basis functions called intrinsic mode functions (IMFs) via an iterative process called “sifting.” IMFs must satisfy two criteria: first, the number of the extrema points (local minima and maxima) and the number of zero crossings must be equal or differ by one at most; second, the mean of the envelopes determined by local extrema points should be zero. The sifting algorithm is executed as follows:

- Identify all extrema of *x* (*t*) and interpolate between the minima *e*_*m*_ (*t*) and maxima *e*_*M*_ (*t*) to find envelope of the signal.
- Compute mean of the envelope, *m*(*t*)=[*e*_*n*_(*t*)+*e*_*M*_(*t*)]/2.
- If *m* (*t*) satisfies the requirements, extract the first “mode” as *x *(*t*) = *x* (*t*) − *φ*_*i*_ (*t*).
- Iterate on the residual *r*(*t*) until it is constant or a trend.

Hence, the original signal can be reconstructed by the sum of the IMDs [31] as described by equation (10) where *L* is the number of IMFs.

(10) $$\begin{array}{c} x\left(t\right)=\sum_{i=1}^{L}\varphi_{i}\left(t\right)+r\left(n\right). \end{array}$$

**Figure 3:**
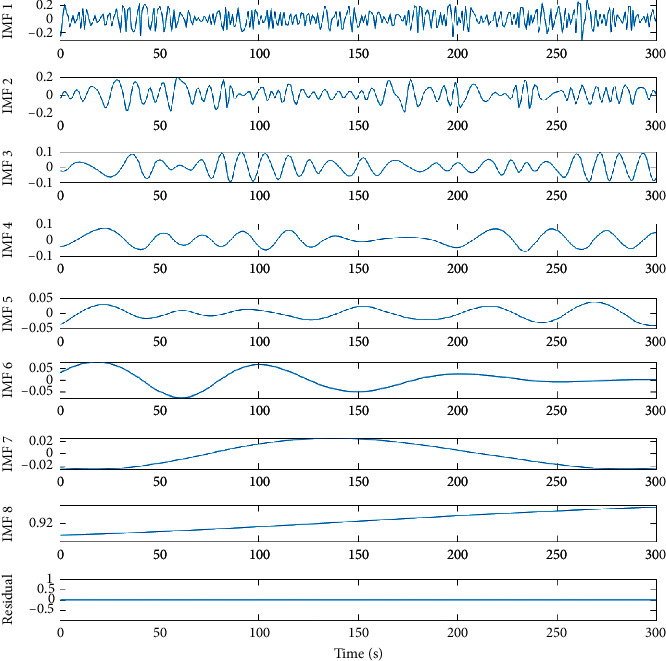
Coarse to fine IMFs obtained from the distorted synthetic signal. Only the first 3 IMFs are used to reconstruct the RR interval tachogram prior to PSD estimation.

Some methods (in more traditional signal processing applications) have proposed the Hurst coefficient as the decision base for which IMF to include in a reconstructed signal [31, 32], but this has specific meaning in the context of HRV analysis [33] and could compromise the meaning of long-range correlations that are used to predict pathological states. Neto et. al. [34] demonstrated that the first three IMFs are sufficient to denoise and recompose the HRV signal in order to analyse LF, HF, and LF/HF content. Following this approach, the RR tachogram is reassembled by summing values from the original time location for the first three IMFs (Figure 3).

### 2.4. Application in “Real” Data

A short portion (for clarity) of data from a single “HD patient” from the iTrend study is used here to illustrate how a different interpretation of the PSD may arise from different preprocessing techniques. Clinically relevant findings from the iTrend population are presented elsewhere [35], but in summary, a total of 50 adult participants were recruited to the study, from which 43 participants had at least one monitored HD session of 4 hours duration following a short interdialytic gap (48-hours). The mean age was 61.5 ± 16.6 years, 26 (60.5%) were male, and 19 (44.2%) had diabetes. The median duration since dialysis therapy was initiated was 24 months (IQR 75), and arteriovenous fistula was the predominant dialysis access (83.7%). Data from ECG lead II [18] was sampled at 300 Hz to avoid issues with QRS detection [4] and then further processed offline [17]. All computer codes were implemented in Matlab version 2020a, but it should be noted that built-in functions for the LS Periodogram, EMD, and band-power integrations could not be used (due to the need to specify and test aspects of the algorithms as discussed above). The mean was removed from the RR tachogram prior to application of one of the above preprocessing methods, and the time series was recalculated to preserve synchronicity where required.

## 3. Results and Discussion

### 3.1. Performance and Limits of Lomb–Scargle Periodogram

**Figure 4:**
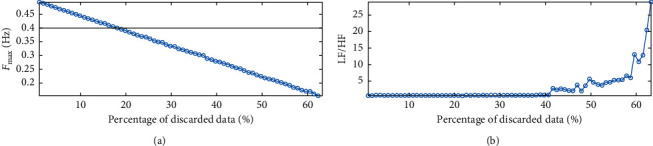
Illustration of the effect of reducing the number of points in the LS periodogram estimate. The lower pane shows that the LF/HF ratio is relatively well estimated using only 41% of the available data, but the window limit is reached when 20% of the data is discarded, shown in the upper pane.

In order to explore the effects of missing data on the LS periodogram, two initial tests were performed where data were discarded from the synthetic signal without noise (Figure 1) and where noise was added to the signal without discarding data. In the first test, the number of discarded data points was increased in 1% increments until the point where there were insufficient data points remaining to perform a spectral analysis of the signal. This occurred when 64% of the RR intervals were discarded and is a function of the grid spacing (Δ*f*=1/5*T*), i.e., data corresponding to a particular frequency bin is missing from the tachogram.  Figure 4 shows that as an increasing number of data points are excluded, the maximum frequency of the signal is reduced. The upper bound of *F*_max_ = 0.4 Hz is reached when only 20% of the data is discarded from a 5-minute window. This agrees with findings that the PSD for time-invariant data series becomes distorted when more than 20% of the data is in error or corrected [27].

The LS periodogram was more robust to increasing amplitudes of noise and was able to successfully locate the LF and HF peaks at a FAP of 1% even when the noise was scaled to have four times the amplitude of the RR tachogram signal (standard deviation of 0.896), confirming the choice of LS for clinical settings. In combination, the maximum level of distortion that can be applied on the fewest number of points corresponds to a SNR ratio of 13.4 dB with 20% of data discarded at random (Figure 2). This maximally distorted signal was them used in further tests to evaluate the effect of signal processing techniques.

In order to establish some criterion against which the effects of signal preprocessing can be compared, the original synthetic signal of equation (4) is compared against the maximally distorted signal in Table 1 where the mean of 100 simulations are presented. These simulations used 100 different additive, zero mean Gaussian white noise profiles that were scaled to ensure the signal had a consistent standard deviation of 0.224 ± 0.03 and SNR of 13.4 dB ± 0.5 dB, using random seeds that were generated from the state of the computer [16]. The addition of noise has increased the total power of the signal by an average of 63% leading to a similar increase of power in both the LF (69%) and HF (65%) bands, and calculation of the 3 dB widths show that both peaks are less narrowly defined in the noisy signal.

The LS periodogram accurately locates both peaks within a very noisy signal but is better able to locate the HF peak corresponding to the sine wave with the larger (2.5 bpm) amplitude. This is consistent with the application of equation (6) leading to the lower mean-average RR interval and a greater number of shorter RR intervals (∼326) and hence a greater emphasis of the HF band.

The RR tachogram is an unusual signal with time represented on both axes, so it may seem surprising that the frequency is reliably identified but amplitude is not. This effect can be seen by comparing the calculation of the LF/HF ratio against its theoretical value from the amplitude of the sine waves is (*A*_*l*_/*A*_*h*_)^2^ ≈ 0.64. This value is closely approximated in the original synthetic signal (Figure 1), but not the distorted signal (Figure 2). The LS approach effectively assumes a sinusoidal model for the RR tachogram data, and so the periodogram height at any frequency is related to how well the model fits the data. The noisy periodogram is based on a more complex model (sinusoids plus white noise, with coincidental alignment of spurious peaks), and so the periodogram is higher at all frequencies, not just those of interest. As a simple example, a calculation of LF/HF ratio of the distorted signal on the basis of normalised units, decibels, and linear units (ms^2^/Hz) results in values of 0.52 ± 0.09, 1.146 ± 1.2, and 1.15 ± 0.03, respectively. The most consistent approach for a signal obtained in a clinical setting that may contain missing data is to express LF and HF in normalised units as percentages of total power between the limits of 0.04 and 0.4 Hz [12].

FFT and AR methods are known to suffer distortion to the resulting power spectrum by leakage due to the implicit rectangular window, and this effect has been explored and discussed elsewhere [12, 13, 36]. In these results, the fraction of power in the main lobe (within ± 0.01 Hz) of each peak shows some evidence that the LS periodogram exhibits a small amount spectral leakage which predominantly occurs in the LF band.

### 3.2. RR Editing with Synthetic Data Distorted by Noise

In order to investigate the effects of signal processing, the 100 different Gaussian white noise profiles were superimposed onto the original synthetic signal (Figure 1) generating 100 different RR tachograms. RR intervals were identified as suspect where they exceeded a specified upper and lower threshold (+32.5% or −24.5%, respectively) [28] and these suspect intervals were edited using the five different procedures defined above. On average, 15% of the RR intervals were classified as in error (5% too long, 10% too short). A summary of data obtained from 100 simulations is presented in Table 2, which shows that it was not possible to fully recover the statistical properties of the original RR tachogram (Figure 1), but all methods were able to replicate the statistical properties of the “distorted” signal (Figure 2) with close agreement in terms of mean RR intervals. The range value can be understood as describing the degree to which the original signal has been smoothed by preprocessing methods. Those methods specifically derived for equidistant time-series spectral analysis (“Rules” editing and RR replacement) were the least able to locate the LF and HF peaks above a FAP of 10%, nor were these peaks located at the correct frequency.

**Figure 5:**
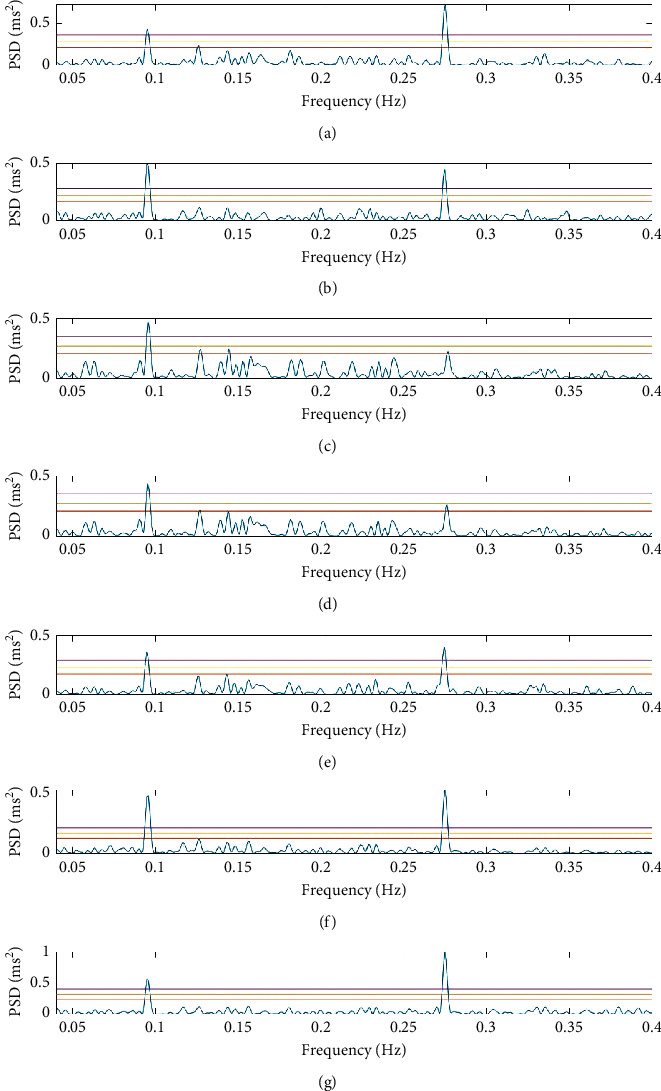
PSD estimation via the LS periodogram using the same original data series and symmetric RR interval selection (±10%), but with five different methods of editing dubious RR intervals. Methods 1 (“baseline”), 2 (“rules”), 4 (OUGP), and 5 (EMD) are considered successful as they were able to identify the LF and HF peaks corresponding to the sine waves in (3). Methods where dubious RR intervals were replaced by cubic spline interpolation, linear interpolation, or by the mean average of one minute of data are considered unsuccessful as background peaks are emphasised and HF peaks are lost in the noise. FAP levels are indicated by the three horizontal lines on each graph, with the top (purple) line showing a FAP of 1%, the mid (yellow) line showing a FAP of 10%, and the lower (orange) line showing a FAP of 50%. (a) Lomb–Scargle baseline. (b) Rules edited RR intervals. (c) Cubic spine interpolation. (d) Linear interpolation. (e) Mean average replacement. (f) OUGP. (g) EMD.

A common feature of all methods (except by EMD) is that both the theoretical (*A*_*l*_/*A*_*h*_)^2^ and actual LF/HF ratios were consistently overestimated (Table 2). This is thought to be related to the asymmetrical basis used to identify aberrant RR intervals. The smaller tolerance on the lower bound biases the identification of aberrant RR intervals, which results in a greater number of HF components being excluding or edited from the PSD estimate. The use of a symmetrical basis of a similar magnitude (±10%) was tested using the same 100 simulations, and the results (presented in Table 3) show an improved agreement between *a*_*l*_/*a*_*h*_ and LF/HF ratios. In real data, any data point is as likely as the next to be in error and all attempts to identify aberrant RR intervals are likely to induce errors. Both OUGP and EMD apply to all data points in the time series and show a clear advantage in this respect. On the basis of this result, a symmetric selection criterion is recommended and will be used throughout the rest of this paper (Figure 5).

Method 1 (“baseline”) involves the simple exclusion of suspect RR intervals from the periodogram estimate and results in a closer approximation of the spectral parameters than methods 2 (“rules”) and 3 (replacement) that attempt to correct information in the RR tachogram by editing and replacing suspect values (Tables 2 and 3). This is a significantly different outcome to previous work based on time-invariant methods where exclusion of RR intervals leads to a systematic loss of information [27].

It is surprising how poorly method 2 performed given that the rules are based on sound physiological principles. The HF peak exceeds a FAP of 10% in only 89% of the simulations, which means that the largest amplitude sine wave forming the synthetics signal was most frequently lost. The PSD estimate had one of the lowest total powers, suggesting that method 2 removed a significant amount of all data without discriminating well between noise and signal. Closer inspection of the corrected RR interval tachogram shows that “rules” based editing offers no improvement over replacement methods whenever errors occur in clusters as noise peaks were emphasised and merged with the true peaks, resulting in an inconsistent prediction of power in the LF and HF bands (Figure 5).

The smoothing effect seen in method 2 (“rules”) is apparent in method 3, regardless of the particular technique used to replace RR intervals. Replacement leads to the poorest estimation of spectral parameters; power in the HF band is attenuated with the replacement methods acting (in effect) as low-pass filters that emphasise local trends (Figure 5). All replacement methods are particularly poor where aberrant RR intervals occur in clusters as the correction becomes arbitrary. None of these approaches can be recommended in conjunction with the LS periodogram.

Method 4 (OUGP) suffered a similar loss of information from the HF band, although it outperformed the “rules” method in terms of accurate location of the peaks, and both LF and HF peaks exceeded FAP of 10% in 100% and 97% of simulations, respectively. OUGP leads to the “smoothest” PSD with the least variation in RR intervals, which is unsurprising in that it applies to all data points and not just suspicious ones. It offered no improvement in estimation of spectral parameters over the “baseline” (method 1) approach.

EMD (method 5) outperforms all others in terms of preserving the statistical features of the underlying signal and was the only method able to consistently locate the HF peak in all 100 simulations. Noise peaks never exceed the FAP threshold of 50% suggesting that this would be the most robust method for preprocessing clinical data. EMD applies to all data points in the RR tachogram and is the only method to accurately estimate the power in the LF band. It also yields the closest prediction of LF/HF ratio. The PSD estimate has a higher power content than the “baseline” (method 1), which suggests that some noise is decomposed into the first three IMFs. It is the subject of future work to establish means by which this could be refined. The challenge of understanding the physiological basis of each IMF (and therefore applying more elaborate denoising approaches [37]) also remains.

### 3.3. RR Editing with Synthetic Data with Physiological Artefacts

The previous section explores the limits of the LS periodogram where a periodic signal is masked by noise. The presence of physiological artefacts leads to a different kind of distortion to the RR tachogram and is considered separately here. In particular, the presence of ectopic beats is known to distort the spectral analysis leading to the guidance that five-minute intervals containing ectopic beats should not be analysed [3, 13]. Within a population prone to cardiac dysrhythmia such as HD patients, this condition could result in no five-minute segments of data being suitable for spectral analysis, even over the course of a four-hour treatment. This experiment is intended to be representative of data processing issues that occur when real patient data is used and where it might be unclear whether the resulting PSD is valid.

Patient data obtained in a clinical setting is hard-won, and so it is a worthwhile exercise to understand whether limitations established for equidistant time series methods also apply to the LS periodogram, such that data are not excluded unnecessarily, and to establish limits on the confidence that an investigator might have in the resulting analysis. To this end, a series of RR intervals in the distorted tachogram (Figure 2) were replaced with two types of false beats that represent typical errors seen in ECG data for patients receiving HD. The first kind represent short beats where an individual RR interval was replaced by one of 200 ms duration (representing a false trigger, such as a tall *T* wave or an ectopic beat), and the second represents missed triggers where two successive RR intervals were replaced by a single longer RR interval of equal duration (representing a missed trigger). The number of false beats was incremented until the point where the PSD estimates fail—a failure was defined as the point where a spurious peak exceeds a FAP of 50% or else where the true peak falls below a FAP of 50%. False beats were tested both as individual occurrences and as part of an increasingly long error burst. Once again, suspect RR intervals were identified by a deviation of ±10% from the previous RR interval.

This set of experiments is summarised in Table 4, which compares the same five different approaches to preprocessing the RR tachogram, with the addition of one further test where spectral analysis is performed on all data in the RR tachogram, including the false beats, to provide a comparison (labelled as LS All Points). Cubic spline interpolation (CSI) was used to represent the replacement methods as it appears to be the most commonly used in literature [8].

The question as to whether preprocessing is necessary is answered by considering the results of the application of the LS method using all points in the five-minute segment. Here the PSD estimate is better able to accommodate discrete errors but is significantly influenced by clusters of errors to the point where it fails when as few as 2 false beats are combined. Its particular mode of failure is to induce spurious peaks into the PSD estimate, which merge with background noise. These peaks and their aliases tend to distort the HF content of the signal and appear as strong periodic content. By comparison, the most robust method involves the identification and exclusion of suspect RR intervals (“baseline”) resulting in a reliable PSD estimation until the window limit was reached (a function of the granularity of the distorted RR tachogram).

The performance of “rules” based editing is dependent on the particular rule in operation. Where a false beat is detected, it is either divided or combined with neighbours or both, and this smoothing process tends to reduce the prominence of false beats within the tachogram, allowing the true peaks in the PSD to be preserved. The peaks are also preserved when long chains of missed triggers are replaced by an equivalent number of RR intervals with the mean average duration, but only a maximum of 6 ectopic beats in an error burst can be processed before failure. The “rules” method tends to reduce differences between RR intervals, and this method fails when smoothing causes the HF peak to disappear. CSI has the opposite mode of failure and introduces false peaks above a FAP of 90% with the inclusion of the first false beat. Most of the noise in the PSD appears in the LF band which merges with the true periodic content, and CSI emphasises this effect as false shapes.

OUGP and EMD apply to all data points in the RR tachogram including false beats and both preprocessing methods allow successful location of periodic signals. The mode of failure for both methods is that the background noise increases in magnitude and/or true peaks decrease in magnitude as the number of false beats increased until the true peaks are lost below a FAP of 50%. OUGP increasingly filters HF content, whereas EMD flattens the whole power spectra.

Table 4 records the values of LF, HF, and LF/HF in the final successful PSD estimation before each method fails. While both peaks are correctly located at a magnitude greater than a FAP of 50%, spectral parameters can be unreliable at this point. The method of correction will always bias the PSD estimated in the direction of the edited RR interval—for example, if a large number of ectopic beats are replaced by a smaller number of long RR intervals, HF power will be reduced, and LF/HF will be overestimated. This is an important issue in analysing a time series of patient data, where successive data segments may contain different types of false beats and different patterns of corrections. For example, the magnitude of the spectral parameters predicted by the “rules” method varies greatly as false beats are increased as the values depend on which rule is deployed for a given condition.

EMD and the “baseline” method provide the most consistent and stable values for spectral parameters as the number of false beats increase, but the latter tends to underpredict HF content leading to an over prediction in LF/HF. OUGP shows a falling trend in HF power leading to a rising trend in LF/HF calculation and is particularly poor when missed triggers (long RR intervals) are included in the tachogram.

All of this leads to a fairly complicated outcome; where a signal is masked by noise, both EMD and OUGP provide a reliable means of locating the periodic components hidden within a signal. Where the RR tachogram is complicated by missed triggers leading to unusually long RR intervals or very short ectopic beats, the more reliable approach is to simply exclude false beats from the PSD estimate. However, neither EMD nor OUGP can operate with missing data points, so it raises the question of whether preprocessing methods could be combined. As EMD operates by locating zero crossings within the RR tachogram, suspect points can be replaced by linear interpolation between neighbouring minima and maxima points to effectively exclude them from the resulting IMF. It is unclear how each IMF is related to physiological processes, and so it is unclear how editing would change the meaning of the PSD, so it is avoided here. As OUGP is a filter, the equivalent method to omitting a suspect beat is to replace it with the mean average, but this would come with the penalty of smoothing the HF content further attenuating power in the HF band.

The distortion of the PSD estimate will depend on the type of distortion in the RR tachogram, the frequency of the periodic content within the HRV signal (and the aliases of these frequencies), the location of suspect RR intervals within a local trend, and whether or not corrected RR intervals merge with noise peaks leading to false peaks. The only sensible recommendation is that whatever method of RR editing is chosen should first be tested with synthetic data to understand its limitations and likely biases prior to analysis of real data.

### 3.4. RR Editing with Patient Data

HRV signals can be highly dynamic and are further complicated by the effects of haemodialysis [14]. This can lead to difficulty in matching sampling rates or processing techniques to the signal, with the result that it is nearly impossible to establish which features of the PSD estimate are real and which are artefacts. To this end, an example of patient data is evaluated to identify further issues with PSD estimate and establish a basis for evaluating results.

**Figure 6:**
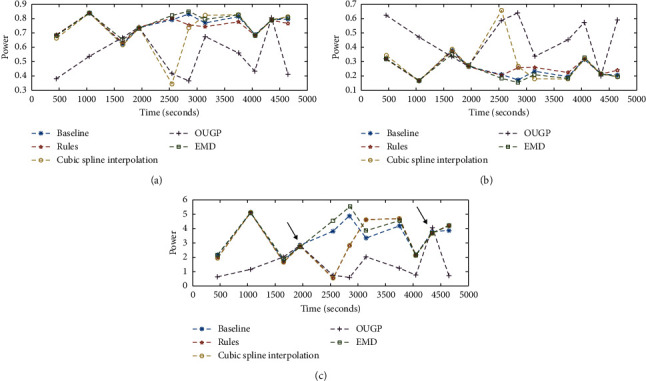
Comparison of spectral parameters over the first 80 minutes (a short time duration is presented for clarity) of haemodialysis. Data points are centred within valid five-minute segments extracted from the RR interval tachogram, with the same segment being preprocessed by five different methods (CSI and “rules” based editing methods are included for illustration as their distorting effects are less apparent with real data). Only two PSD estimates (4^th^ and 10^th^) are based on RR tachograms without any suspect data points, and these are shown by the arrows in the lower pane. (a) LF (nu) results during HD treatment. (b) HF (nu) results during HD treatment. (c) LF/HF (nu) results during HD treatment.

Figure 6 presents results of spectral analysis from only the first 80 minutes of an uncomplicated four-hour long HD treatment involving a patient that is considered to be haemodynamically stable (at 80 minutes, this patient took a tea break, providing a natural end point for this truncated and illustrative example). The SNR of this dataset is estimated to be 10.4 dB, and the standard deviation of the signal is 0.228 over the 80-minute duration, providing a more difficult test than the distorted signal used previously. In other words, these data represent the most straightforward RR tachogram that is likely to be obtained in a clinical setting. This 80-minute record of data could theoretically generate 16 five-minute segments of data; however, only 11 segments met previously defined criteria: two segments were rejected for having fewer than 240 data points within the sample; two segments generated PSD estimates that had no peaks above a FAP of 50%, and so any periodic content was completely masked by noise; the final rejected segment fell short of the window limit where missing data caused *f*_max_ < 0.4 Hz.

Within the remaining 11 segments of data, identification of suspect RR intervals was based on the symmetric criteria of ±10% deviation from the previous RR interval [7]. This identifies 8% of all RR intervals as being in error (with 7% being too short and 1% too long). None of the individual 11 segments contained more than 10% RR intervals identified as suspect, and as before, suspect data points were excluded from the PSD estimate in the “baseline” case, corrected prior to spectral analysis for “Rules” and CSI methods, and included without correction for EMD and OUGP.

To simply exclude any five-minute segment that contains suspect data would result in little to no information being available for analysis. If that were done here for a well-behaved dataset, only 2 PSD estimates would remain (these being the fourth data point centred at 1950 seconds, and the 10^th^ at 4350 seconds indicated by arrows in Figure 6) which show a close but not identical estimation of spectral parameters between all methods. These “correct” estimates indicate that a slight rising trend in LF/HF is observed during the first 60 minutes of dialysis, which clearly illustrates a problem with the behaviour of the OUGP method. This approach to identify trends based only on error-free segments can be helpful within large datasets to rapidly screen for suspect PSD estimates.

Figure 6 also shows that the application of these different preprocessing methods leads to considerable diversity in estimation of LF and HF power and highlights a significant problem that may occur when comparing results from different studies. The estimation of spectral parameters by alternative methods can lead to significantly different results even for the same dataset. The use of a single PSD estimate from a fixed point in dialysis, which can deviate significantly from an overall trend, could therefore be an unreliable indicator of patient response further complicating comparisons and compromising its diagnostic significance.

**Figure 7:**
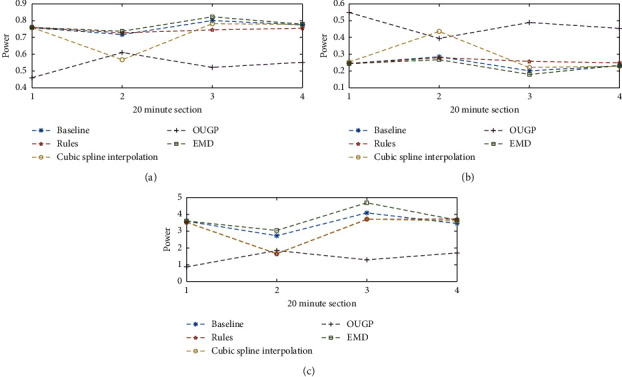
Each point represents the mean-average of spectral parameters (derived from valid PSD estimates) over a 20-minute period. It presents the results from the first 80 minutes of a dialysis treatment, and it can be seen that different trends arise from different methods of processing the RR tachogram. (a) 20-minute average of LF results. (b) 20-minute average of HF results. (c) 20-minute average of LF/HF results.

Given the variability in the results, any attempt to mediate fluctuations by averaging spectral parameters is also likely to be unreliable.  Figure 7 presents the results from averaging spectral parameters over 20-minute intervals. OUGP shows a sharp rise in LF/HF immediately after the start of dialysis, and CSI and “rules” methods show a sharp fall. In clinical studies, this pattern would be associated with different outcomes. Low LF has been associated with intradialytic hypotension [38], and sharp falls would characterise this patient as being haemodynamically unstable [5] which they are not. OUGP tends to return higher levels of HF power. This behaviour could also be a serious failing in clinical studies, where reduced HF band power is associated with worse patient outcomes [1, 21]. In this case, the artificially high levels could mask an underlying morbidity.

**Figure 8:**
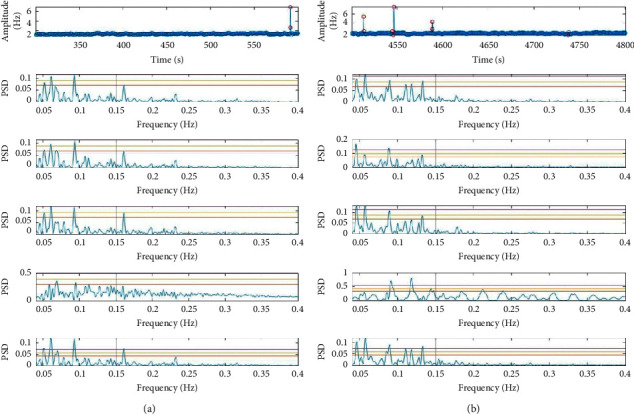
(a) Effect of a single short beat. (b) Effect of multiple errors.

Figures 6 and 7 also show that OUGP tends to follow a different trend to the other methods and returns the most varied results. This suggests that using a consistent method of evaluating results does not necessarily result in a consistent output. CSI and the “rules” method are generally in agreement but tend to apportion different levels of power to the LF and HF bands, with CSI being much more susceptible to the effects of ectopic beats. EMD and the “baseline,” both previously identified as the most reliable, also show close agreement. These differences are better explained with reference to the PSD estimates presented in Figure 8.

Comparison of RR editing methods using data from a “HD patient.” The RR tachogram is shown in the top panes, with suspect intervals identified by red markers for clarity. The same RR interval tachogram is processed by five different methods shown in the panes below. These data correspond to the first and last time-series points in Figure 6.

The presence of extremely short duration RR intervals in Figure 8 has been caused by an artefact derived from the processing the ECG signal where a portion of the signal was masked by a short burst of noise, probably caused by patient movement. The “rules” method suggests that these two short intervals in Figure 8(a) should be replaced by adding them to the previous RR interval, while CSI replaces these beats with a single interpolated value—both corrections are plausible, and visual inspection of the ECG trace offers no guidance as to which is correct. A single, discrete error leads to only a small difference in spectral parameters and small (but visible) changes to the PSD.  Figure 8(b) shows a series of suspect RR intervals, and in this case, the “rules” method corrects them as more, shorter duration RR intervals while CSI corrects to fewer, longer duration intervals resulting in noticeably different PSD estimates. As the correct correction is uncertain, the advantage of the “baseline” approach is evident in that these suspect data points are simply excluded from the calculation. The operation of EMD is similar in that it treats the “ectopic” peak as belonging to a long period sine wave that appears in a higher order IMF and therefore is not used to reconstruct the signal prior to PSD estimation. The only difference between the two methods is scale with the “baseline” PSD appearing as an attenuated version of EMD. It should be noted that as the number of erroneous data points increases, the “baseline” method is increasingly less able to locate any peaks above a FAP of 50%.

Figure 8(a) shows something is clearly amiss with OUGP, which helps illustrate perhaps the most crucial test of the validity of the results—consideration of the source of the pattern of the peaks revealed by the LS periodogram. Multiple peaks exceeding a FAP probability of 50% can be seen in the LF band for the other five methods, these being LF peaks associated with rhythmic changes in vascular tone, baroreceptor response, and respiratory sinus arrhythmia [13, 27]. A single peak in the HF band associated with respiration occurs just after 0.15 Hz in all examples, except for OUGP where it falls well below a FAP of 50%. The loss of these peaks clearly shows that the OUGP method has failed.

The mechanism of this failure is more evident in Figure 8(b), where a greater number of data points are in error. The PSD generated from OUGP identifies two significant peaks in the LF band; the first (just before 0.1 Hz) is apparent in all of the estimates, while the second is not. OUGP applies to all of the erroneous data points and decomposes some of this noise into the signal prior to LS estimation, which highlights a common mode of failure in spectral analysis approach—the largest peaks might not be real. Moreover, OUGP exaggerating HF components is unexpected given the performance of this method with synthetic data. Closer inspection of Figure 8(b) shows that the inclusion of the “noise” leads to the OUGP filter saturating, and so *m*-order harmonics of the two LF peaks appear throughout the HF band (*m* can be 2 or 3). Spurious peaks could also be caused by some unexpected aliasing effect in the window function, but there is no good way to deconvolve the window from the signal in LS periodogram estimation to test this effect.

In summary, OUGP is not sufficiently robust to be used with clinical data and both CSI and “rules” based methods lead to different PSD estimates without any clear way to establish which is correct. The most reliable method is to exclude suspect points (as with the “baseline”); however, the effect of excluding suspect data points is to attenuate the signal and risks of true peaks being lost when HRV signals have very low amplitudes (such as for very sick or elderly patients). In the case of clinical data, EMD is the more reliable method for dealing with suspect RR intervals but with much more strict limits on the maximum number of errors that can be included. The experiments conducted in the previous section suggest that a maximum 8 discrete ectopic beats, or 4 within an error burst, can be safely processed by EMD accommodated without distorting the results.

## 4. Discussion

The calculation and analysis of PSD for HRV studies is not trivial. Two distinct but overlapping processes generate highly dynamic short-term responses, these being the complex relationship between the sympathetic and parasympathetic nervous system and the regulatory mechanisms of heart rate, blood pressure, and baroreflex in response [23]. As the RR interval tachogram represents time on both axes, it cannot discriminate between the actuation and response effects.

Nevertheless, studies of HRV can reveal significant and important diagnostic and prognostic information, both about the patient as an individual [38] and as part of a population [1]. Abnormal HRV primarily reflects the dysregulation between sympathetic and parasympathetic nervous system and has been associated with an increased risk of morbidity [1]. Within populations receiving HD treatment, a low degree of HRV indicates impaired autonomic function and a reducing HRV has been associated with adverse cardiovascular outcomes [39]. Comparisons of HRV taken before and after HD have also proved to be a useful clinical marker in predicting overall mortality [2].

Given the significance of these findings, it is surprising that HRV does not have greater diagnostic use. There may be two reasons for this; the first would relate to the time involved in manually inspecting ECG traces and RR interval tachogram prior to analysis, and the second is in the variability of results [27] which has been shown here to arise from the method of correcting errors in addition to any underlying physiological basis—both of which make interpreting the results more difficult.

This work attempts to address both aspects by demonstrating that the LS periodogram provides a reliable and robust estimate of PSD even in clinical (as opposed to research) conditions, provided that proper attention is given to the frequency limits and sampling grid's role in suppressing or exaggerating spurious peaks in the PSD (this may address criticisms regarding the overly spiky appearance of the LS PSD estimate [13]). In summary,

- The window limit (*F*_max_) provides a hard upper limit and is a function of the sampling frequency. It is more important than pseudo-Nyquist frequency when unevenly sampled data are analysed.
- Violation of the lower-limit condition leads to an attempt to analyse power below the fundamental frequency limit of the signal. For this reason, total power should be calculated between 0.03 Hz and 0.4 Hz to avoid boundary effects (lumping) when integrating the PSD from 0 to calculate the normalised LF and HF values. This effect cannot be assumed to be small.
- The lowest number of points that can be analysed in a 5-minute segment is 240, leading to a lower limit of 48 bpm on heart rate [12].

A consistent recommendation in literature is that five-minute segments of data containing more than 20% of suspect or edited RR intervals should not be used for HRV analysis [3, 23], which holds true for the LS method. However, a four-hour HD treatment could generate 48 PSD estimates per patient per treatment, and so the use of FAP provides an important screening criterion to understand whether a five-minute segment is valid for analysis. Its use here has demonstrated that methods employed to edit the RR tachogram via smoothing techniques are unsuitable in conjunction with the LS periodogram. Smoothing methods (in general) act as low-pass filters, emphasising local trends and filtering high-frequency components of the signal. The different use of the RR editing method could explain some of the contradictory results that appear in literature. Kuo et al. [40] associated a lower LF/HF ratio with better survival for patients receiving HD and noted that while some studies agreed with this result [2] others found lower LF/HF ratio had more adverse ESRD events and poorer survival [41]. Kuo et al. [40] discussed the differences in terms of study design and clinical factors, but it is also possible that the different methods used PSD estimation which could also have influence the results. For example, Chen et al. [2] who reported a positive association with lower LF/HF ratios estimated PSD from five minute segments of data where attempts were made to attenuate spectral leakage by using a Hamming window. Brotman et al. [41] applied FFT to 2-minute segments of data that were filtered and smoothed and reported a negative association with lower LF/HF ratios.

The investigation performed here also contradicts previous findings that the method of RR interval selection has a greater effect on PSD than the method of RR editing [7]. The major issue seems to be one of bias caused when the method of RR interval selection is asymmetric. The results here suggest that selection criteria should always be symmetrical when used with the LS periodogram, whether a mean average, confidence interval, or previous RR interval provides the basis of comparison.

Attempts to edit RR intervals using “rules” based on physiological plausibility is not helpful. In reality, any RR interval is equally as likely to be in error as its comparator. If editing is required, it should be applied to the whole time series. The recommendation from this work is that EMD is the preferred technique for denoising. Where the data are subject to artefacts— particularly missing data and ectopic beats, the LS “baseline” method is the most robust. Suspicious RR intervals should always be excluded from the LS PSD estimate rather than edited, and the use of a synthetic data series to probe the limits of the chosen processing technique is valuable. This initial test should allow PSDs containing small number of artefacts, including ectopic beats, to be processed without compromising the integrity of the analysis. If this is done, then it should be noted that overall trends in data are more reliably identified using the PSD estimate arising from error-free segments of the RR tachogram. PSD estimates that deviate from such trends can then be easily identified and further investigated.

Other criticisms of HRV analysis note that spectral parameters derived from five-minute intervals do not have the prognostic power of time-domain measurement derived from 24 hours of data [23], and yet spectral parameters are used (sometimes successfully [2]) to predict long-range outcomes. It is possible that a more deliberate approach to PSD estimation could lead to better correlation between the two time periods.

## 5. Conclusions

The LS periodogram for spectral analysis seems to be highly suitable for use with patient data obtained in a clinical setting as it is more robust to noise effects than other methods and is able to work with missing data points and artefacts including ectopic beats. However, its application requires some deviation from guidelines established for the more common time invariant methods used to estimate PSD.

When using the LS periodogram to estimate spectral parameters of heart rate variability, it is more appropriate to exclude data points than to edit them. The basis for the identification of suspicious RR intervals will lead to identification of a greater or fewer number and has no further effect provided the method is symmetrical. Should further preprocessing be necessary, EMD is the preferred method for denoising.

The LS periodogram estimates can only be made when maximum and minimum frequency limits are observed and where the grid spacing is derived from sampling frequency for each five-minute interval. These could be dynamic within a single time series.

The use of synthetic data to establish the limits of the processing technique is recommended, and results should be interpreted in light of these results. A further check of whether the features of the PSD estimate can be related to physiologically plausible mechanisms and effects can provide additional confidence in the results, aiding comparison between studies.

Finally, calculation of FAP should always be performed in deciding whether to accept the PSD estimate of five-minute segment as valid. This decision point can enable greater automation and therefore greater clinical use of the analysis.

## Figures and Tables

**Table 1:** Results from 100 simulations of random noise in the “distorted” signal (Figure 2) compared against the ls periodogram estimated for the original synthetic signal (Figure 1).

	Original synthetic signal	Distorted signal (mean ± std)
Mean average RR interval (sec)	1.009 ± 0.0038	0.9196 ± 0.0061
Range RR intervals	0.151	0.870 ± 0.110
LF power (nu)	0.39	0.32 ± 0.04
HF power (nu)	0.61	0.62 ± 0.04
LF/HF	0.6357	0.52 ± 0.09
LF peak height (ms^2^/Hz)	0.1668	0.54 ± 0.16
HF peak height (ms^2^/Hz)	0.2601	0.68 ± 0.18
(*A*_l_/*A*_*h*_)^2^	0.6413	0.79 ± 0.34
LF peak location (mHz)	95	97.4 ± 19.6
HF peak location (mHz)	275	275.8 ± 9.1
LF Δ*f*_3dB_ (Hz) ×10^−3^	3.00	3.6 ± 0.11
HF Δ*f*_3dB_ (Hz) ×10^−3^	2.90	3.4 ± 0.91
Fraction of power within ± 0.01 Hz of LF band	0.76	0.69 ± 0.21
Fraction of power within ± 0.01 Hz of HF band	0.75	0.86 ± 0.22
Total power (dB)	−28.46	−17.97 ± 0.41

All data points in the distorted signal are used to estimate the power spectra.

**Table 2:** Comparison of results obtained from five different methods of RR editing (using 100 simulations with different random noise profiles) and asymmetric selection criteria (+32.5% or −24.5%).

	Method 1	Method 2	Method 3 (replacement)			Method 4	Method 5
	“Baseline”	“Rules”	Cubic spline	Linear	Mean	OUGP	EMD
Mean RR (sec)	0.94 ± 0.01	0.93 ± 0.01	0.94 ± 0.01	0.94 ± 0.01	0.94 ± 0.01	0.91 ± 0.01	0.92 ± 0.01
Range RR (sec)	0.64	0.72	0.74	0.73	0.73	0.59	0.81
LF (nu)	0.39 ± 0.04	0.41 ± 0.05	0.43 ± 0.05	0.44 ± 0.05	0.38 ± 0.04	0.43 ± 0.04	0.32 ± 0.04
HF (nu)	0.51 ± 0.04	0.48 ± 0.05	0.45 ± 0.04	0.43 ± 0.04	0.52 ± 0.04	0.55 ± 0.04	0.66 ± 0.04
LF/HF	0.77 ± 0.13	0.87 ± 0.18	0.97 ± 0.18	1.05 ± 0.19	0.75 ± 0.13	0.8 ± 0.13	0.48 ± 0.09
Total power (dB)	−19.08 ± 0.38	−19.49 ± 0.45	−18.42 ± 0.4	−18.87 ± 0.4	−19.59 ± 0.39	−19.57 ± 0.47	−17.97 ± 0.45
LF location (mHz)	95.0 ± 1.52	91.8 ± 15.38	72.5 ± 30.38	68.3 ± 31.28	91.0 ± 24.4	96.0 ± 0.92	94.9 ± 0.31
HF location (mHz)	273.1 ± 17.96	257.2 ± 53.17	156.3 ± 81.28	140.8 ± 74.37	248.2 ± 56.28	263.1 ± 46.44	275.0 ± 0.29
(*A*_*l*_/*A*_*h*_)^2^	1.15 ± 0.52	1.72 ± 0.78	1.33 ± 0.91	1.29 ± 1	1.52 ± 0.75	1.97 ± 0.89	0.75 ± 0.29
% of LF peak > FAP 10%	97	93	78	80	92	100	99
% of HF peak > FAP 10%	97	89	81	84	79	97	100

Data are presented as mean ± std.

**Table 3:** Comparison of results obtained from 5 different methods of RR editing (using 100 simulations with different random noise profiles) and symmetrical selection criteria of ±10%.

	Method 1	Method 2	Method 3 (replacement)			Method 4	Method 5
	“Baseline”	“Rules”	Cubic spline	Linear	Mean	OUGP	EMD
Mean RR (sec)	0.92 ± 0.01	0.92 ± 0.01	0.92 ± 0.01	0.92 ± 0.01	0.92 ± 0.01	0.91 ± 0.01	0.92 ± 0.01
Range RR (sec)	0.67	0.74	0.75	0.74	0.74	0.59	0.85
LF (nu)	0.38 ± 0.05	0.41 ± 0.04	0.41 ± 0.05	0.43 ± 0.05	0.38 ± 0.05	0.43 ± 0.04	0.32 ± 0.04
HF (nu)	0.52 ± 0.04	0.5 ± 0.04	0.48 ± 0.04	0.45 ± 0.04	0.53 ± 0.04	0.55 ± 0.04	0.62 ± 0.04
LF/HF	0.74 ± 0.14	0.83 ± 0.15	0.88 ± 0.17	0.96 ± 0.19	0.73 ± 0.14	0.79 ± 0.14	0.52 ± 0.09
Total power (dB)	−18.07 ± 0.41	−19.29 ± 0.44	−18.08 ± 0.39	−18.51 ± 0.39	−19.21 ± 0.4	−19.51 ± 0.49	−17.64 ± 0.43
LF location (mHz)	96.5 ± 14.37	94.3 ± 5.18	75.4 ± 37.93	73.2 ± 36.96	92.2 ± 25.19	95.0 ± 0.36	95.0 ± 0.31
HF location (mHz)	273.3 ± 16.34	267.8 ± 32.14	165.9 ± 83.31	163.9 ± 85.46	249.7 ± 59.68	275.0 ± 0.28	275.0 ± 0.28
(*A*_*l*_/*A*_*h*_)^2^	1.13 ± 0.57	1.68 ± 0.65	1.32 ± 0.9	1.31 ± 0.98	1.44 ± 0.76	1.21 ± 0.49	0.78 ± 0.3
% of LF peak > FAP 10%	97	93	78	80	92	100	99
% of HF peak > FAP 10%	97	89	81	84	79	97	100

Data are presented as mean ± std.

**Table 4:** Location of the maximum number of false beats that can be included in a five-minute segment before failure, with LF, HF, and LF/HF (n.u.) recorded in the final “successful” PSD estimate.

Method	Ectopic beats (individual)				Ectopic beats (group)				Missed trigger (individual)				Missed trigger (group)			
	Max. false beats	LF/HF	LF	HF	Max. false beats	LF/HF	LF	HF	Max. false beats	LF/HF	LF	HF	Max. false beats	LF/HF	LF	HF
True value		0.47	0.29	0.63		0.47	0.29	0.63		0.47	0.29	0.63		0.47	0.29	0.63
“Baseline”	23	0.55	0.32	0.58	35	0.56	0.32	0.58	20	0.57	0.33	0.57	32	0.64	0.35	0.55
“Rules” edited RR intervals	17	0.64	0.33	0.52	6	0.61	0.32	0.53	18	0.69	0.34	0.49	16	0.76	0.36	0.47
CSI	0	0.77	0.38	0.50	0	0.75	0.37	0.50	1	0.78	0.39	0.50	1	0.75	0.38	0.50
OUGP	4	0.45	0.31	0.68	4	0.64	0.37	0.58	7	0.75	0.42	0.56	2	0.89	0.45	0.51
EMD	8	0.28	0.22	0.76	4	0.50	0.32	0.65	9	0.62	0.37	0.60	4	0.45	0.29	0.66
LS (all points)	7	0.34	0.24	0.70	2	0.37	0.25	0.68	3	0.38	0.25	0.66	2	0.52	0.31	0.61

## Data Availability

Data are available on request from the corresponding author.
